# Cross-Parallel Transformer: Parallel ViT for Medical Image Segmentation

**DOI:** 10.3390/s23239488

**Published:** 2023-11-29

**Authors:** Dong Wang, Zixiang Wang, Ling Chen, Hongfeng Xiao, Bo Yang

**Affiliations:** College of Engineering and Design, Hunan Normal University, Changsha 410081, China; 202120183241@hunnu.edu.cn (D.W.); 202270183474@hunnu.edu.cn (Z.W.); lcheno@hunnu.edu.cn (L.C.); 18374972137@163.com (H.X.)

**Keywords:** medical image segmentation, parallel ViT, MHSA, activation function

## Abstract

Medical image segmentation primarily utilizes a hybrid model consisting of a Convolutional Neural Network and sequential Transformers. The latter leverage multi-head self-attention mechanisms to achieve comprehensive global context modelling. However, despite their success in semantic segmentation, the feature extraction process is inefficient and demands more computational resources, which hinders the network’s robustness. To address this issue, this study presents two innovative methods: PTransUNet (PT model) and C-PTransUNet (C-PT model). The C-PT module refines the Vision Transformer by substituting a sequential design with a parallel one. This boosts the feature extraction capabilities of Multi-Head Self-Attention via self-correlated feature attention and channel feature interaction, while also streamlining the Feed-Forward Network to lower computational demands. On the Synapse public dataset, the PT and C-PT models demonstrate improvements in DSC accuracy by 0.87% and 3.25%, respectively, in comparison with the baseline model. As for the parameter count and FLOPs, the PT model aligns with the baseline model. In contrast, the C-PT model shows a decrease in parameter count by 29% and FLOPs by 21.4% relative to the baseline model. The proposed segmentation models in this study exhibit benefits in both accuracy and efficiency.

## 1. Introduction

Medical image segmentation is a vital research area due to its distinct properties that differentiate it from RGB image segmentation. Its significance in medical applications further highlights its necessity. The encoder–decoder structure, based on the Convolutional Neural Network (CNN), was a pioneering aspect of this field [1,2]. It exhibited excellent receptive fields and contextual information in the deep layers of the network, making it adaptable to multiscale input images. Additionally, it represented an end-to-end training model that received significant attention at the time. This innovation gave rise to the foundational U-Net framework [3], based on the “U”-shaped network structure, sparking a wave of research enthusiasm. The U-Net network structure is characterized by its simplicity, featuring a fully symmetric encoder–decoder architecture with skip connections. Due to its outstanding network performance, it has dominated the field of medical image segmentation. However, CNNs with local receptive fields have limitations in extracting global features for tasks with long-term relationship dependencies, making them unable to fully capture global information. This restricts the CNN’s ability to realize its full potential.

Recently, network models based on the Transformer architecture [4] have been challenging the dominant position of CNN and gaining prominence, primarily due to their self-attention mechanism, which possesses the capability to model long-range contextual information. This addresses the limitations of CNNs, making them shine in the field of medical imaging. The concept of incorporating Transformer modules into the network architecture of U-Net has reignited a research wave centered around Transformer-based approaches in the domain of medical image segmentation. On one hand, most researchers have been exploring how to embed serial Transformer modules into the U-Net structure, leading to a series of classic networks such as TransUNet [5], Swin-Unet [6], UNETR [7] and so on [8,9,10,11,12]. Undeniably, serial Transformer network models have significantly improved the accuracy of medical image segmentation. Notably, the TransUNet [5] model was the first to apply the Vision Transformer (ViT) [13] to the field of medical image segmentation, leveraging the global contextual modeling capabilities of Transformers in conjunction with the local feature extraction characteristics of CNN. This has provided highly effective solutions for the medical domain. It is evident that their competitive advantage has been achieved through increased model complexity, which inevitably comes with high computational and memory costs, potentially impacting the practical application of these models in clinical medical segmentation [14]. On the other hand, there has been relatively less research on the application of parallel Transformer modules in medical image segmentation [15,16,17]. This is because traditional parallel modes tend to increase network parameters and feature dimensions, which can impact network efficiency and accuracy. However, the emergence of parallel ViT [18] has provided a new direction for applying parallel Transformers to medical image segmentation research. Under the condition of maintaining the same parameter count, replacing serial ViT [13] with parallel ViT can increase network width while reducing network depth. Parallel ViT achieves this by reducing the depth of the modules, optimizing network training, and making network training less challenging compared with serial ViT. However, despite maintaining the same parameter count, it still incurs high computational costs. Additionally, the reduction in network depth weakens its semantic representation and contextual awareness, limiting the applicability of parallel ViT in medical image segmentation. Therefore, there is a need for an effective parallel structure that can simultaneously enhance the accuracy and efficiency of medical image segmentation, breaking the limitations of parallel ViT applications.

In this research, we reevaluated the design of the parallel ViT structure, addressing its shortcomings in semantic representation and the issue of high computational costs. We improved and optimized a classic end-to-end network, TransUNet [5], and introduced the Cross-Parallel Transformer module (C-PT block) to address these challenges. We replaced the serial ViT block in TransUNet with the parallel ViT and C-PT block, naming them PTransUNet (PT model) and C-PTransUNet (C-PT model), respectively. Specifically, we first enhanced TransUNet by incorporating the parallel ViT. Without changing the model’s parameter count and FLOPs, this approach aimed to reduce the optimization complexity of the network. The goal was to accelerate global context modeling on top of rich local features, leading to better feature representation. Next, we improved the proposed C-PT block in TransUNet, leveraging semantic information fusion and cross-attention from the left and right branches of the parallel Transformer. This enhancement strengthened the long-range modeling capabilities and high-order spatial interactions of the parallel ViT in a collaborative manner to obtain more robust feature representations. Furthermore, in order to reduce computational expenses and minimize memory usage, we streamlined the Feed-Forward Network (FFN) [19] architecture within the parallel ViT, replacing it with the Rectified Linear Unit (ReLU) activation function. This overall enhancement improved the segmentation performance. We conducted experiments that compared the performance impact of parallel Transformer structures applied to the TransUNet network model from the perspectives of depth and parallelism. We assessed the model’s segmentation accuracy and efficiency, and through ablation experiments, we analyzed the effectiveness of the C-PT block design.

The contribution of this paper consists of what follows:

- We propose an enhanced PTransUNet model by utilizing parallel ViT rather than serial ViT in the TransUNet medical image segmentation model. This model demonstrates an improvement in segmentation efficiency in comparison to the TransUNet model.
- We present an enhanced C-PTransUNet model and introduce a cross-parallel Transformer module to upgrade the parallel ViT. The MHSA part employs the Dendrite Net (DD) [20,21] layer to enhance semantic representation and remote spatial dependencies. It achieves self-crossover attention for semantic features through feature fusion. In the Feed-Forward Network section, we employ a “rude” streamlining approach. This entails the removal of the fully connected layer and using exclusively the combination of normalization and activation functions. This results in reduced computational overhead while preserving the nonlinear feature representation.
- On the public dataset Synapse [22], we experimentally evaluate the PT model and C-PT model, and the findings demonstrate that parallel ViT has superior accuracy and efficiency than sequential ViT for medical image segmentation.

## 2. Related Work

### 2.1. Vision Transformers Development

The ViT model, which was first designed for Natural Language Processing, has now been successfully applied to Computer Vision. It competes favourably with conventional CNN approaches in tasks including image classification, target detection, and semantic segmentation. Its success is attributable to its dynamic attention mechanism and long-range modelling capabilities, which prove its robust feature learning. The image is divided into multiple small patches by ViT. These patches are then turned into sequences that serve as input features. An N-layer Transformer processes these sequences to produce a thorough feature representation of the entire image. With the self-attention mechanism, the Transformer captures long-distance dependencies among image features and enables higher-order spatial information exchange. It excels in global relational modeling, expanding the receptive field and acquiring rich contextual details, which effectively complements the global modeling capabilities of CNN.

Recently, a variety of novel models based on the ViT backbone network have been born, which may be categorized into sequential and parallel Transformer architectures. The sequential ViT models include DeiT [23], CeiT [24], Swin Transformer [25], T2T-ViT [26], PVT [27], DeepViT [28], and others. As the Transformer module is concerned with global contextual information, it is concerned with building relationships between pixels throughout the whole image range. It is unable to capture local visual features like standard CNN utilizing inductive bias, increasing the difficulty of ViT training and delayed convergence. Touvron et al. [23] proposed the DeiT model, which attempts to learn the inductive bias of image data and distill the knowledge utilizing the teacher model CNN, which is then passed to the student model Transformer, which may enhance feature extraction via convolutional bias and also accelerates model convergence. For sequence input, ViT will partition the input image into numerous patch blocks; these fixedly divided patch blocks will lose the image’s local features. The Swin Transformer [25] model adopts the idea of dynamic attention to neighbouring pixels, using sliding windows to model globally in the spatial dimension, while performing self-attention operations in each patch block and attention computation across blocks; this dynamic generation of attention weights reduces the computational complexity of self-attention and improves local feature extraction. Each ViT Transformer layer has the same resolution image characteristics, resulting in a high computational cost. Yuan et al. [26] proposed a T2T-ViT model which utilizes a deep and narrow hierarchical Transformer architecture to enhance the features but at a high computational cost. Wang et al. [27] suggested a Pyramid Vision Transformer (PVT) model with an asymptotically shrinking feature pyramid hierarchical structure that can acquire multi-scale feature maps and an attention layer SRA to reduce the computational consumption of processing high-resolution feature maps. DeepViT [28] is designed with Re-Attention, which re-performs the self-attention operation with multiple features across layers at a cheaper computational cost, relieving the deep ViT feature saturation problem and allowing the network to learn more complicated representations.

Currently, serial Transformer structure research is thriving, but parallel Transformer structure research is limited [16,17,29]. Parallel ViT [18] is the first improvement proposed by the Meta team, in which the serial connected Transformer blocks are converted to parallel processing by decreasing the depth of the model while increasing the width of the model, and the residual portion becomes smaller as the network becomes deeper, and the parallel processing can be approximated to be equivalent to the sequential ViT [13]; at this time, the number of model parameters and FLOPs are not changed. Depth [30,31] and width [32] are two critical factors for neural network architecture. To boost performance, most ViT variants [23,24,25,26,27,28] increase the depth by concatenating the Transformer. Deep networks are difficult to optimize, and the model’s separability is affected by the size of the feature dimension. There are fewer studies on ViT width expansion now [18], and the main concern is that parallel ViT raises the computational cost of the network, increases the model’s complexity, and the feature dimensions are too high to be easily overfitted.

### 2.2. Transformer-Based Medical Image Segmentation Method

Replacing the convolutional block of the U-network with a Transformer module capable of global feature extraction is a promising avenue to investigate the application of the Transformer for medical image segmentation. The TransUNet [5] was the pioneer network model implementing the ViT for medical image segmentation. As CNN captures only local information, embedding the Transformer in the codec to extract global features of the CNN image-coding block can acquire long-distance model dependencies and rich spatial information. In order to achieve accurate segmentation, the decoder up-samples the coded features and performs feature localization with CNN low-level features. The TransUNet model incorporates the self-attention mechanism into the U-Net architecture to enhance contextual comprehension, but this comes with a notable computational expense. The Swin-Unet [6] model, inspired by the Swin Transformer [25] module, replaces the U-Net network’s convolutional layer directly, leading to the first pure Transformer structure for medical image segmentation. The input image undergoes a non-overlapping patch operation before being fed into the Transformer encoder to learn the global deep feature representation. The decoder then combines the encoded features with up-sampled features to recover the feature map and perform segmentation prediction. This approach resolves the issue that convolution struggles to learn global semantic information effectively. UNETR [7] aims to convert the 3D segmentation task into a sequence-to-sequence prediction problem. This model’s encoder learns semantic features over long distances using a pure Transformer architecture, while the decoder retrieves high-resolution features with a CNN structure. UNETR uses a hybrid Transformer-CNN approach because it recognizes that ViT, even though it is excellent at extracting global features, does not perform well in acquiring local semantic information, and that the Transformer has a greater computational overhead than CNN. As previously mentioned, conventional network architectures including TransUNet [5], Swin-Unet [6], and UNETR [7] utilize ViT or Swin Transformer modules to enhance feature extraction through the increase of network depth (i.e., the series connectivity pattern of the blocks [33]). It is evident that increasing the network depth has a greater impact on model performance, yet this may not be the ideal selection when considering network optimization, separability, and computational costs.

The proposed Cross-Parallel Transformer (C-PT) module aims to improve model performance while reducing computational overhead and solving existing issues. By better balancing depth and parallelism choices, the C-PT module achieves these goals. In this study, we verify the module’s effectiveness on the classical 2D medical image segmentation network TransUNet. Furthermore, this work represents a courageous attempt to apply parallel Transformer to medical image segmentation. To illustrate the Transformer’s connection patterns, Table 1 lists several conventional networks. In particular, PTransUNet is crafted with parallel ViT, and the suggested C-PT block is fortified with parallel ViT. We revised a portion of the ViT structure and examined a more efficient Transformer connection. Our results indicate that this modification improves TransUNet, even though only a fraction of its architecture is altered.

## 3. Methods Section

### 3.1. Cross-Parallel Transformer Module

**Figure 1:**
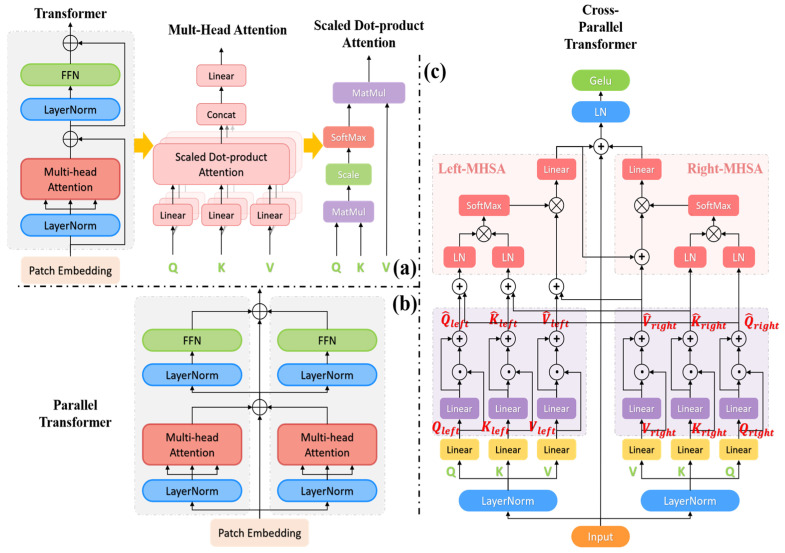
(**a**) Transformer module. (**b**) Parallel Transformer module. (**c**) Cross-Parallel Transformer module. QKV represents the Query-Key-Value in the self-attention mechanism. After passing through the linear layers and the DD layer in the left and right layers, the QKV is respectively mapped to ${\hat{Q}}_{*}$ , ${\hat{K}}_{*}$ , and ${\hat{V}}_{*}$ . * represents the left block or the right block.

In this paper, we propose a cross-parallel Transformer module (C-PT), as illustrated in Figure 1. The module applies parallel design principles to enhance image feature extraction and improve image segmentation accuracy by increasing its width. As opposed to the conventional design of the Transformer module, this paper presents a cross-parallel transformer module comprising the cross-parallel multi-head self-attention block (C-PMHSA) and an activation function block. The C-PMHSA incorporates the multi-head self-attention residual block (MHSA) of the conventional Transformer module. At the same time, the multilayer perceptron (MLP) within the standard Transformer module is simplified as an activation function block. The connection composition is very concise, retaining only the Layer Normalization (LN) and GELU [34] activation functions. The key aim of this proposal is to fully utilize the Transformer’s parallel nature, improve the multi-head self-attention mechanism’s feature extraction ability [35], simplify the FFN structure in the latter stages, and minimize computational complexity without compromising module performance.

#### 3.1.1. Cross-Parallel Multi-Head Self-Attention Blocks (C-PMHSA)

We have revised both the internal structure and connection of the two parallel MHSA modules to create a new cross-parallel MHSA structure. This new structure defines the parallel structure as left-branching and right-branching Transformer blocks, known as left and right blocks.

The image features are linearly normalized once in each of the left and right blocks, before being mapped to the query matrix ( $Q_{*}$ ), key-value matrix ( $K_{*}$ ), and value matrix ( $V_{*}$ ) of the corresponding blocks through the linear transformation matrices $W_{Q\_*}$ , $W_{K\_*}$ , and $W_{V\_*}$ of the left and right blocks, respectively. These matrices are calculated as follows:

(1) $$Q_{*}=W_{Q_{\_*}}\left(LN_{*}\left(x\right)\right),$$

(2) $$K_{*}=W_{K\_*}\left(LN_{*}\left(x\right)\right),$$

(3) $$V_{*}=W_{V\_*}\left(LN_{*}\left(x\right)\right),$$

where $LN_{*}$ denotes the operator for layer normalization, with * representing the left or right blocks, and x signifying the position-encoded input block for the image.

Unlike the conventional Transformer module, we introduce a DD layer [20], i.e., a white-box model, after the $W_{Q\_*}$ , $W_{K\_*}$ , and $W_{V\_*}$ linear layers in the left and right blocks. The DD layer proceeds through a linear layer before conducting a Hadamard product operation with the input; its primary aim is to enhance the nonlinear representation of the model by emulating dendritic processing in neurons. In order to avoid excessive computational complexity and enhance the model’s generalization ability, we propose incorporating a single layer of Dendrite Net after the left and right blocks. While the Hadamard product operation of the DD layer can suppress low-interest regions in the image features, it may also diminish high-interest features. In this regard, we addressed the feature loss issue resulting from the DD layer by incorporating residual connections [36] and implemented proximity connections to reinforce the acquisition of highly relevant features. The mapping matrices of the left and right blocks are calculated as follows:

(4) $${\hat{Q}}_{*}=W_{Q\_*}^{10}Q_{*}\circ Q_{*}+W_{Q_{*}}^{10}Q_{*},$$

(5) $${\hat{K}}_{*}=W_{K\_*}^{10}K_{*}\circ K_{*}+W_{K\_*}^{10}K_{*},$$

(6) $${\hat{V}}_{*}=W_{V\_*}^{10}V_{*}\circ V_{*}+W_{V\_*}^{10}V_{*},$$

where $W_{Q\_*}^{10}$ , $W_{K\_*}^{10}$ , and $W_{V\_*}^{10}$ represent the weight matrices of the DD layer, ∘ indicates the Hadamard product of the DD layer, $Q_{*}$ , $K_{*}$ , and $V_{*}$ denote the original feature inputs of the DD layer, and * denotes the same as above.

Subsequently, the feature information from both the left and right blocks is combined in the left block before undergoing the multi-head self-attention operation. This involves adding the mapping matrices ${\hat{Q}}_{*}$ , ${\hat{K}}_{*}$ , and ${\hat{V}}_{*}$ of both blocks to maximize the utilization of the extracted image features, improving feature expression capability and reducing feature loss. The corresponding mapping matrices ${\hat{Q}}_{*}$ and ${\hat{K}}_{*}$ of the left and right blocks undergo a LN to reduce data redundancy and hasten convergence. The attention input matrix is computed as follows:

(7) $${\hat{Q}}_{left}^{\prime }={\mathrm{LN}}_{Q\_left}\left({\hat{Q}}_{left}+{\hat{Q}}_{right}\right),$$

(8) $${\hat{K}}_{left}^{\prime }={\mathrm{LN}}_{K\_left}\left({\hat{K}}_{left}+{\hat{K}}_{right}\right),$$

(9) $${\hat{V}}_{left}^{\prime }={\hat{V}}_{left}+{\hat{V}}_{right},$$

(10) $${\hat{Q}}_{right}^{\prime }={\mathrm{LN}}_{Q\_right}\left({\hat{Q}}_{right}\right),$$

(11) $${\hat{K}}_{right}^{\prime }={\mathrm{LN}}_{K\_right}\left({\hat{K}}_{right}\right),$$

where ${\mathrm{LN}}_{Q\_left}$ , ${\mathrm{LN}}_{K\_left}$ , ${\mathrm{LN}}_{Q\_right}$ , and ${\mathrm{LN}}_{K\_right}$ are layer normalization operators.

In this paper, the multi-headed self-attention operations within the left and right blocks maintain the QKV computation method as introduced in the regular Transformer module [4]. This involves mapping the matrices Q, K, and V to distinct subspaces for attention computation, while maintaining the same number of parameters. The attention results of the distinct subspaces are then spliced together to capture the correlations between the image blocks. In particular, the left block operation involves a self-attention calculation, whereas the right block operation involves a self-cross-attention calculation. In this case, the input matrices ${\hat{Q}}_{left}^{\prime }$ , ${\hat{K}}_{left}^{\prime }$ and ${\hat{V}}_{left}^{\prime }$ for the left block attention computation incorporate the feature fusion information from the left and right blocks, whilst primarily concentrating on their individual contextual features. The input matrices ${\hat{Q}}_{right}^{\prime }$ and ${\hat{K}}_{right}^{\prime }$ used for the right block attention computation contain feature information from this layer. Meanwhile, the input matrix ${\hat{V}}_{right}^{\prime }$ represents the fusion of the left block output matrix $O_{left}$ and the right block mapping matrix ${\hat{V}}_{right}$ . This resulting matrix guides the generation of highly correlated image features by the right block using the image feature information from the left block. Similarly, this paper retains the Dropout [37] layer after softmax and MHSA to reduce the adaptation between all neurons and address potential overfitting issues. Additionally, the output from the left and right blocks are fused for feature alignment to prevent feature loss, then connected to the input with residuals. This step aims to mitigate the training difficulty of the network with gradient degradation. The left block’s self-attention ( ${\mathrm{Att}}_{left}$ ) and output ( $O_{left}$ ) are defined as follows:

(12) $${\mathrm{Att}}_{left}\left({\hat{Q}}_{left}^{\prime },{\hat{K}}_{left}^{\prime },{\hat{V}}_{left}^{\prime }\right)={\mathrm{Drop\_soft}}_{left}\left(\mathrm{softmax}\left(\frac{{\hat{Q}}_{left}^{\prime }{\left({\hat{K}}_{left}^{\prime }\right)}^{T}}{\sqrt{d_{k}}}\right)\right){\hat{V}}_{left}^{\prime },$$

(13) $${\mathrm{head}}_{left}^{i}={\mathrm{Att}}_{left}\left({\hat{Q}}_{left}^{\prime }W_{i}^{Q},{\hat{K}}_{left}^{\prime }W_{i}^{K},{\hat{V}}_{left}^{\prime }W_{i}^{V}\right),$$

(14) $${\text{MHSA}}_{left}\left({\hat{Q}}_{left}^{\prime },{\hat{K}}_{left}^{\prime },{\hat{V}}_{left}^{\prime }\right)=\text{Concat}\left({\text{head}}_{left}^{1},\ldots ,{\text{head}}_{left}^{h}\right)$$

(15) $$O_{left}={\mathrm{Drop\_att}}_{left}\left({\hat{W}}_{left}{\mathrm{MHSA}}_{left}\right),$$

where Equations (13) and (14) can be located in reference [4].

The right block’s self-crossing attention ( ${\mathrm{Att}}_{right}$ ) and output ( $O_{right}$ ) are defined as follows:

(16) $${\hat{V}}_{right}^{\prime }={\hat{V}}_{right}+O_{left}$$

(17) $${\mathrm{Att}}_{right}\left({\hat{Q}}_{right}^{\prime },{\hat{K}}_{right}^{\prime },{\hat{V}}_{right}^{\prime }\right)={\mathrm{Drop\_soft}}_{right}\left(\mathrm{softmax}\left(\frac{{\hat{Q}}_{right}^{\prime }{\left({\hat{K}}_{right}^{\prime }\right)}^{T}}{\sqrt{d_{k}}}\right)\right){\hat{V}}_{right}^{\prime },$$

(18) $${\mathrm{head}}_{right}^{i}={\mathrm{Att}}_{right}\left({\hat{Q}}_{right}^{\prime }W_{i}^{Q},{\hat{K}}_{right}^{\prime }W_{i}^{K},{\hat{V}}_{right}^{\prime }W_{i}^{V}\right),$$

(19) $${\text{MHSA}}_{right}\left({\hat{Q}}_{right}^{\prime },{\hat{K}}_{right}^{\prime },{\hat{V}}_{right}^{\prime }\right)=\text{Concat}\left({\text{head}}_{right}^{1},\ldots ,{\text{head}}_{right}^{h}\right)$$

(20) $$O_{right}={\mathrm{Drop\_att}}_{right}\left({\hat{W}}_{right}{\mathrm{MHSA}}_{right}\right),$$

where Equations (18) and (19) refer to the same as above.

#### 3.1.2. Activation Function Block

The regular Transformer’s FFN segment features the conventional MLP connection, comprising fully connected layers and activation functions. It undergoes a process of upscaling and then downscaling the matrix dimension and achieves significant global feature interaction, but has a heavy computational overhead. The Transformer module [4] is crucial for the global nature of MLPs. However, in vision MLPs, only spatial location features are perceived globally [38], and channels are not interacted with. The self-attentive mechanism compensates for this limitation by utilizing the information between channels, thereby allowing global features to interact attentively. In this paper, the C-PMHSA is proposed as a replacement for the MHSA in the conventional Transformer. This enhancement improves the feature extraction capability of the multi-head self-attention mechanism [35], albeit at the cost of higher computational effort. To alleviate computational pressure in the C-PT module, this paper suggests removing the computationally expensive MLP in favour of a simple activation function block. The activation function block, which omits fully connected layers, consists of LN layers and the GELU activation function. GELU has the ability to smooth out input distributions [34], thereby alleviating gradient vanishing issues and enhancing model training efficiency.

### 3.2. Improvements to the TransUNet Model

The TransUNet model is a medical image segmentation network that utilizes the Transformer module, unique in its kind. Consequently, the model delivers comparable segmentation results in 2D medical image segmentation. Its encoder employs a hybrid CNN-Transformer architecture, wherein certain CNN blocks in the U-Net encoder have been partially replaced by Transformer blocks. This move cleverly reduces the computational cost resulting from the large input matrix typical of the pure Transformer module. Our proposed C-PT module is an evolutionary outcome derived from ViT, which attributes its triumph to three factors: (1) image segmentation instead of pixel-level inputs [13], (2) a multi-head self-attention mechanism [4], and (3) the GELU activation function [34]. Since the TransUNet model employs a sequential ViT architecture that is challenging to optimize and computationally costly, its effect on the segmentation accuracy and efficiency of the network model is significant.

**Figure 2:**
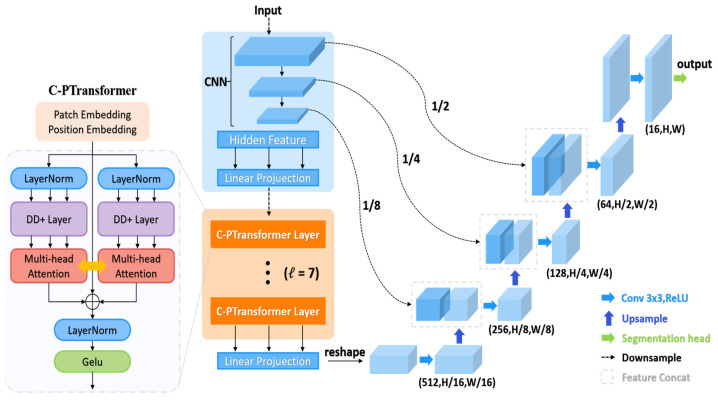
C-PTransUNet Network Architecture. Replacing the sequential Transformer layer within a TransUNet model with a parallel Transformer layer results in the construction of a PT network model. On the other hand, substituting it with a cross-parallel Transformer layer establishes a C-PT network model.

Therefore, we propose two refined models: (i) the PTransUNet network model, which substitutes the sequential ViTs of the TransUNet model with parallel ViTs, thereby preserving the other network structures and not altering the parameter count and FLOPs of the sequential ViTs, and (ii) the C-PTransUNet network model, which replaces TransUNet’s sequential ViT with C-PT blocks. In particular, each layer of the C-PT block also utilizes two MHSAs for feature extraction in the C-PMHSA. Compared with TransUNet, our proposed network model can be expanded in depth. Increasing the number of layers of C-PMHSA can improve feature extraction, benefiting the model’s ability to perform long-range modelling and global spatial interaction. All other aspects of the model will retain the original structure and parameters of the TransUNet model. Figure 2 depicts the network structure of C-PTransUNet. The expression of layer ℓ ’s output is as follows:

(21) $$z_{\ell }^{\prime }=\;C-\mathrm{PMHSA}\left(z_{\ell -1}\right)+z_{\ell -1},$$

(22) $$z_{\ell }=\;\mathrm{GELU}\left(\mathrm{LN}\left(z_{\ell }^{\prime }\right)\right),$$

where $z_{\ell }$ represents the image feature of layer ℓ in the encoder.

## 4. Experiments and Results

In this section, we conducted experiments using publicly available dataset for medical image segmentation tasks. We evaluated the findings through both qualitative and quantitative perspectives and investigated the scalability of the module in ablation experiments.

### 4.1. Dataset

To validate the model, we selected the datasets used in the TransUNet model, namely the Multi-Organ CT Image Segmentation Dataset for the MICCAI Multi-Atlas Abdominal Labelling Challenge (Synapse) [22] and the MRI Image Segmentation Dataset for Automated Cardiac Diagnostics Challenge (ACDC) [39]. Synapse consists of CT scans of eight abdominal organs from 30 cases, comprising a total of 3779 cross-sectional slices. We partitioned the dataset into a training set comprising 18 cases, amounting to 2211 slices, and a test set including 12 cases, consisting of 1568 slices, as per the standard dataset division. For the 8 abdominal organ segmentation tasks, covering the aorta, gallbladder, left kidney, right kidney, liver, pancreas, spleen, and stomach, we present the Dice similarity coefficient (DSC) and the 95% Hausdorff distance (HD95). The ACDC dataset includes cardiovascular magnetic resonance images from 100 cases, which altogether comprise of 1902 cross-sectional slices. We have divided this dataset into three subsets: a training set that includes 70 cases (1300 slices), a validation set of 10 cases (186 slices), and a test set that contains 20 cases (416 slices). We focused primarily on displaying the DSC for the right ventricle (RV), left ventricle (LV), and myocardial layer (MYO).

### 4.2. Implementation Details

Our experiments were conducted using the PyTorch framework with a single NVIDIA A5000 GPU that has 24 GB of memory. To ensure objective comparison with the baseline TransUNet, we applied the same data augmentation technique as used in the TransUNet model to prevent overfitting. We also set the appropriate input resolution (224 × 224, 320 × 320) and patch size P = 16. The same optimizer and parameters [5], comprising a learning rate of 0.01, momentum of 0.9, weight decay of 1 × 10^−4^, etc., were used for training the model. Based on the TransUNet model, we set the batch size to 24 and the number of training iterations to 14 k for the Synapse dataset [5]. Under the stipulation of maintaining the initial requirements, we preserved the pre-training parameters of ResNet-50 [36] concerning ImageNet [40] in the TransUNet design. For the 12-layer Transformer component’s encoder part, we substituted it with a C-PT block with the most suitable number of layers. We employed some of the ViT pre-training parameters to enhance training effectiveness. Following this, we completed general training to adjust the network weights. We additionally utilized 2D inputs for forecasting, followed by the reconstruction of the model in 3D for assessment of the impact. In particular, all Synapse experiments with 512 input image sizes in this paper were performed with a batch size of 6 and a learning rate of 0.0025, which is different from the TransUNet conditions.

Six evaluation metrics were used in our experiment, including the DSC and the HD, in addition to Accuracy, F1 Score, Sensitivity, and Precision. DSC represents the overlap degree between the predicted and labelled images being compared. HD95 represents the largest distance between the first 95% of measures of the anticipated image and the corresponding points of the labelled image. Accuracy assesses the overall correctness of a classification model, whereas the F1 Score can be seen as a harmonic mean of a model’s precision and recall. Sensitivity appraises the proportion of correctly identified instances among the positive class, and Precision gauges the proportion of actual positive instances among the predicted positive samples. Here are the mathematical formulas used to assess performance metrics mentioned previously:

(23) $$DSC=\frac{2*\left|A\cap B\right|}{\left|A\right|+\left|B\right|},$$

(24) $$HD_{95}=max\left\{d_{AB}+d_{BA}\right\},$$

(25) $$Accuracy=\frac{TP+TN}{TP+TN+FP+FN},$$

(26) $$F1\;Score=\frac{2*TP}{2*TP+FP+FN},$$

(27) $$Sensitivity\;\left(Recall\right)=\frac{TP}{TP+FN},$$

(28) $$Precision=\frac{TP}{TP+FP},$$

where *A* and *B* indicate the predicted result and true label regions of the image, respectively, |*A* ∩ *B*| represents the intersection size of these two regions, while |*A*| and |*B*| signify their sizes. $d_{AB}$ is represented by the 95th percentile distance between the predicted outcome and the true labelled value, and $d_{BA}$ is represented by the 95th percentile distance between the true labelled value and the predicted outcome. *TP* is True Positives, *TN* is True Negatives, *FP* is False Positives, and *FN* is False Negatives.

### 4.3. Comparison of Baseline Models

#### 4.3.1. Synapse Dataset Result

Table 2 illustrates the effect of two improved models, PT and C-PT, on the Synapse dataset for multiple abdominal organs, whilst predominantly comparing the assessment outcomes for three distinct input image dimensions.

(1) Input image size of 224: The best performance for our C-PT model was achieved when using the 7-layer C-PT module, with average DSC and HD95 scores reaching 80.73% and 21.15 mm respectively—both surpassing other classical methods. Additionally, it also demonstrated superior segmentation performance compared with the latest CTC-Net [41] and TransDeepLab [42] models. Compared with the baseline model, both of our methods achieve better results. The PT model and the C-PT model outperform the baseline TransUNet [5] by 0.87% and 3.25%, respectively, in terms of average DSC accuracy. Additionally, they shorten the edge gap by 5.31 mm and 10.54 mm in terms of average HD95.

(2) Input image size of 320: The PT model and C-PT model perform marginally better than the baseline method in DSC evaluation metrics, achieving gains of 0.47% and 1.25%, respectively. However, the PT model’s edge prediction is slightly weaker than that of the baseline method in terms of HD, and the C-PT model’s structure is shorter by 2.17 mm than the baseline network.

(3) Input image size to 512: The C-PT model utilizes a 7-layer C-PT module to optimize performance. While the PT model demonstrates an improvement of 0.49% and 1.28% compared to nnUNet [43] and TransUNet [5], respectively, in average DSC metrics, its HD aspect fails to meet desired standards. The C-PT model, on the other hand, exhibits a potential advantage, with its accuracy metric in DSC slightly surpassing that of the baseline method, and a reduction of 4.57 mm in the HD metric. Overall, according to Table 2, our proposed C-PT model demonstrates superior performance in comparison to both the baseline and PT models in overall evaluation metrics. However, the PT model only outperforms the baseline model with regards to the mean DSC. Notably, the C-PT (320 × 320) model is better than the nnUNet (512 × 512) [44] by means of the mean DSC, and also reduces the edge prediction error by 3.63 mm based on mean HD metrics.

Table 3 compares the results of four evaluation metrics in the Synapse dataset. The findings demonstrate that the C-PT model outperforms the baseline model regarding Accuracy, F1 Score, Sensitivity, and Precision, while the PT model is marginally superior to the baseline model, displaying mainly comparable performance.

**Table 2:** Comparative results of mainstream 2D models on the Synapse abdominal multi-organ segmentation dataset. The best results are shown in bold. DSC stands for the higher the better and HD95 for the lower the better.

Methods	Average		Size	Aorta	Gallbladder	Left Kidney	Right Kidney	Liver	Pancreas	Spleen	Stomach
	DSC↑	HD95↓									
V-Net [44]	68.81	-	-	75.34	51.87	77.10	80.75	87.84	40.05	80.56	56.98
DARR [45]	69.77	-	-	74.74	53.77	72.31	73.24	94.08	54.18	89.90	45.96
R50+U-Net [3]	74.68	36.87	-	84.18	62.84	79.79	71.29	93.35	48.23	84.41	73.92
TransClaw UNet [46]	78.09	-	224	85.87	61.38	**84.83**	79.36	94.28	57.65	87.74	73.55
U-Net [3]	78.2	31.96	224	88.31	**70.2**	79.38	71.57	93.75	57.53	86.31	78.52
R50 VIT CUP [13]	71.29	32.87	224	73.73	55.13	75.80	72.20	91.51	45.99	81.99	73.95
CGNET [47]	75.08	-	224	83.48	65.32	77.91	72.04	91.92	57.37	85.47	77.12
AttUNet [48]	75.59	36.97	-	55.92	63.91	79.20	72.71	93.56	49.37	87.19	74.95
Swin-UNet [6]	79.13	21.55	224	85.47	66.53	83.28	79.61	94.29	56.58	**90.66**	76.60
TransUNet [5]	77.48	31.69	224	87.23	63.13	81.87	77.02	94.08	55.86	85.08	75.62
UCTransNet [10]	78.23	26.75	224	-	-	-	-	-	-	-	-
FFUNet [8]	79.09	31.65	224	86.68	67.09	81.13	73.73	93.67	64.17	90.92	75.32
CTC-Net [41]	78.41	22.52	224	86.46	63.53	83.71	80.79	93.78	59.73	86.87	72.39
TransDeepLab [42]	80.16	21.25	224	86.04	69.16	84.08	79.88	93.53	61.19	89.00	78.40
Ours ^1^	78.35	26.38	224	86.65	60.86	82.18	77.50	**94.57**	58.28	89.65	77.12
Ours ^2^	80.73	21.15	224	88.33	65.99	83.84	**82.27**	94.54	63.36	88.28	**79.27**
TransUNet [5]	81.41	23.28	320	90.37	64.98	85.51	**81.60**	94.67	**68.34**	88.41	77.40
Ours ^1^	81.88	30.17	320	89.54	64.90	84.40	80.18	**95.39**	66.27	**91.28**	83.12
Ours ^2^	82.66	21.11	320	89.21	**67.97**	**85.54**	81.18	95.09	66.82	90.79	**84.71**
nnUNet [43]	82.36	24.74	512	90.96	65.57	81.92	78.36	**95.96**	69.36	91.12	**85.60**
TransUNet [5]	81.57	26.89	512	90.45	**66.20**	79.73	74.99	95.25	**74.24**	88.61	83.14
Ours ^1^	**82.85**	28.12	512	**91.01**	63.85	84.38	**80.6**	95.84	70.19	91.61	85.33
Ours ^2^	81.85	**22.32**	512	90.94	60.51	**84.99**	79.38	95.28	68.67	**93.39**	81.71

^1^ Representing the PTransUNet model. ^2^ Representing the C-PTransUNet model.

**Table 3:** Comparative results of baseline and enhanced models on the Synapse dataset. The best results are shown in bold. The better the performance, measured in percentages, follows an increase in values for Accuracy, F1 Score, Sensitivity, and Precision. The input image size is uniformly 224.

Evaluating Indicator	Methods	Average	Aorta	Gallbladder	Left Kidney	Right Kidney	Liver	Pancreas	Spleen	Stomach
Accuracy	TransUNet	99.88	99.97	99.98	99.94	99.93	99.92	99.91	99.90	99.70
	Ours ^1^	99.89	99.97	99.98	99.94	99.93	99.74	99.91	**99.93**	99.73
	Ours ^2^	**99.90**	99.97	99.98	**99.95**	**99.94**	**99.75**	**99.92**	99.92	**99.76**
F1 Score	TransUNet	76.53	87.61	53.24	81.99	76.89	93.76	58.44	86.07	74.24
	Ours ^1^	77.31	86.65	52.53	82.18	77.50	**94.57**	58.28	**89.65**	77.12
	Ours ^2^	**79.69**	**88.33**	**57.65**	**83.84**	**82.27**	94.54	**63.36**	88.28	**79.28**
Sensitivity	TransUNet	76.53	88.36	50.49	**87.67**	73.00	95.04	55.59	90.53	71.56
	Ours ^1^	76.85	87.09	51.62	82.99	76.62	95.81	54.80	**92.01**	73.89
	Ours ^2^	**79.56**	**89.88**	**55.32**	82.47	**83.30**	**95.83**	**60.63**	91.44	**77.66**
Precision	TransUNet	80.29	87.19	62.71	78.58	**83.81**	92.60	73.12	84.51	79.86
	Ours ^1^	80.36	86.72	58.79	81.72	80.16	**93.46**	70.95	**88.54**	82.62
	Ours ^2^	**82.37**	**87.30**	**64.71**	**87.45**	81.94	93.42	**74.80**	86.24	**83.15**

^1^ Representing the PTransUNet model. ^2^ Representing the C-PTransUNet model.

#### 4.3.2. ACDC Dataset Result

Table 4 illustrates the impact of PT and C-PT models on the ACDC cardiac dataset. With regard to average DSC metrics, our C-PT model performs slightly lower than the newest CTC-Net model but displays a potential competitive edge over other models. Our C-PT model outperforms other models in segmenting the Myo region. In a comparison of the same parameter configuration between the TransUNet and C-PT model, the C-PT model exhibited superior segmentation DSC coefficients and HD metrics. Therefore, the results indicated that the C-PT model is more robust.

Table 5 presents the outcomes of the performance evaluation of our model and the baseline model across four performance metrics. The C-PT model surpasses the baseline model in terms of Sensitivity and F1 Score metrics, while slightly lagging behind the baseline model in terms of Precision. The Precision of the PT model was better than that of the baseline model, although it scored slightly lower in terms of F1 Score and Sensitivity. In terms of accuracy, both the C-PT model and the PT model were comparable to the baseline model.

### 4.4. Parallelism Experiment

We examined the influence on performance of varying the numbers of parallel branches on the PT and C-PT models, and contrasted their performance with that of the baseline model [5]. As any parallelism and depth combination changes the number of parameters and FLOPs of the ViT modules, we chose to keep the total number of modules constant for each network model and reallocate modules across different branches. For instance, TransUNet’s sequential ViT-B/12x1 can be combined into PT’s parallel ViT-B/6 × 2 or 4 × 3, 3 × 4. Here, ViT-B stands for the ViT-Base module [13], and 6 × 2, 4 × 3, and 3 × 4 denote 6-layer 2-branching, 4-layer 3-branching, and 3-layer 4-branching, respectively. The layering and branching refer specifically to the module’s depth and degree of parallelism, respectively. The reorganization process does not alter the fundamental parameters of each module [18], such as the Hidden Size and MLP Size. Since the C-PT modules used in the C-PT model are adapted with parallel ViT-B/6 × 2, which has a distinct structure compared to the parallel ViT-B blocks, we regulated how to merge the depth and parallelism of these blocks by simply fixing the total quantity of C-PT modules. The methodology used to combine them is identical to the one used in the PT model, and the C-PT blocks require fewer references and FLOPs than the parallel ViT-B blocks.

**Figure 3:**
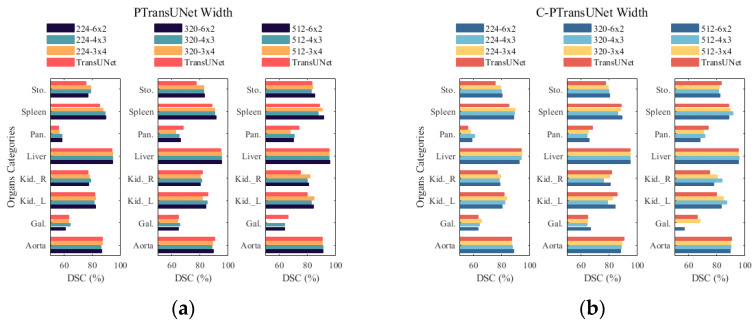
Comparison of DSC for multiple organ segmentation in parallelism experiments. (**a**) Parallelism experiments with different sizes of PTransUNet models; (**b**) Parallelism experiments with different sizes of C-PTransUNet models.

Figure 3 displays the outcomes of three parallelism experiments of the PT and C-PT models conducted on varying image sizes belonging to the Synapse multi-organ segmentation dataset. In Figure 3a, the PT model’s 3 × 4 and 6 × 2 structure proves advantageous over the baseline model for Stomach and Spleen organ segmentation. For Left Kidney and Right Kidney organ segmentation, the PT model’s 4 × 3 and 6 × 2 structure is slightly superior to the baseline model. The overall structure is similar to the baseline model for Liver and Aorta organ segmentation. However, the PT/3 × 4 model exhibits feature redundancy when segmenting 512-sized images, leading to a reduction in the accuracy of Gallbladder organ segmentation. The PT model exhibits variability in segmentation accuracy across organs with variations in parallelism, while the PT/6 × 2 model consistently demonstrates superior segmentation performance across multiple organs compared to the PT/4 × 3 and PT/3 × 4 models. In Figure 3b, it is evident that the segmentation performance of the three parallelisms of the C-PT model improves significantly at an image size of 224. Similarly, the C-PT/6 × 2 model shows comparable results to the baseline for multi-organ segmentation once the input image size is increased to 320. Finally, increasing the image size from 320 to 512 further enhances the performance. The accuracy of the C-PT/4 × 3 and C-PT/3 × 4 models fluctuates during the segmentation of the Stomach, Spleen, Left Kidney, and Right Kidney organs, with varying levels of precision. Furthermore, it should be noted that an increase in parallelism can impact the segmentation stability of the C-PT model.

We adjust the optimal module width of the segmentation network model by varying the level of parallel branching of modules to enhance the segmentation performance. From the experimental data presented in Table 6, it is apparent that the PT model has an equal number of parameters across different modes, including the 6-layer 2-branch, 4-layer 3-branch, and 3-layer 4-branch, with similar FLOPs for identical input image sizes. Interestingly, with input image sizes of 224 and 320, the average DSC of PT/4 × 3 is slightly greater than that of PT/6 × 2 and PT/3 × 4, while the average HD reaches its optimal level at an input size of 320. Increasing the input size to 512, PT/6 × 2 achieves the best performance in terms of average DSC, while it is slightly weaker than PT/3 × 4 in terms of average HD. Although, by increasing the input size, the PT module improves performance at parallelism levels 3 and 4, at 512, the model acquires redundant features, leading to a degradation of the segmentation performance. The performance impact of C-PT models at varying parallelism can be found in Table 7. As the parallel structure in the MHSA component of C-PT is present, the activation function decreases as parallelism increases. Therefore, it becomes apparent that the number of parameters differs between the three models, although the total number of C-PT modules remains the same. Consequently, the FLOPs of all three C-PT models are also the same. Considering the DSC and HD aspects together, it is evident that with an image size of 224, DSC increases as branching increases while HD decreases. In contrast, at an image size of 320, the overall performance deteriorates with an increase in branching. For an image size of 512, C-PT/3 × 4 exhibits superior DSC and HD metrics, as well as a greater number of parameters compared with C-PT/6 × 2. However, due to the higher probability of redundant features and unstable segmentation performance, the increase in parallelism is unsuitable for depth scaling. In summary, considering the segmentation model’s performance and computational overhead, both the PT model and C-PT model in this paper use a degree of parallelism of 2.

### 4.5. Depth Experiment

**Figure 4:**
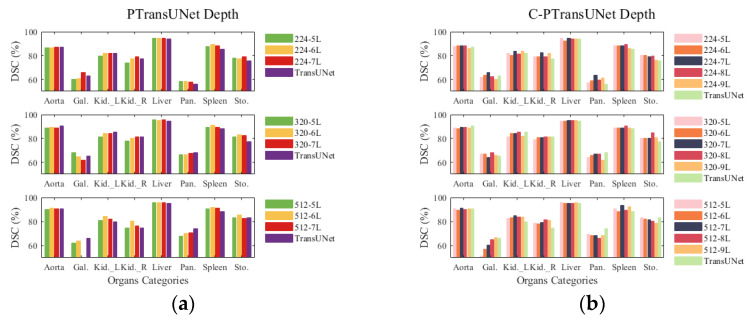
Comparison of DSC for multiple organ segmentation in depth experiments. (**a**) Depth experiments with different sizes of PTransUNet models; (**b**) Depth experiments with different sizes of C-PTransUNet models.

Figure 4 shows the outcome of scaling the depth of the PT and C-PT models for three image sizes using the Synapse multi-organ segmentation dataset, with a parallelism of 2. In Figure 4a, for the PT model used in multiple organ segmentation, its Dice similarity coefficient (DSC) does not exhibit a consistent increase with depth, which is only observed in the cases of 224-Gallbladder, 224-Left Kidney, 224-Right Kidney, 320-Left Kidney, 320-Right Kidney, 320-Pancreas and 512-Pancreas. As the image scale increases, the PT-6L model displays a more pronounced advantage in segmentation accuracy compared with PT models of other depths. Compared with the baseline model, the PT model demonstrates a competitive advantage when the depth is set to 6 (without altering the number of consecutive ViT parameters and FLOPs). In Figure 4b, the C-PT/7L model achieved better segmentation results in multi-organ segmentation as the image scale increased from 224 to 512. It is noteworthy that the C-PT/8L model outperformed the C-PT/7L model in the segmentation of Gallbladder, Left Kidney, Spleen, and Stomach organs at an image scale of 320. The C-PT model may enhance segmentation performance through enhancing the depth of appropriate modules.

To investigate how variations in module depth affect the performance of PTransUNet (PT) and C-PTransUNet (C-PT) networks, we adjust the depth by expanding and compressing the number of layers. This is based on the results obtained from previous experiments to determine the optimal number of parallel branches. Our main focus is the performance analysis of the PT network model at depths ranging from 5 to 7 layers and the C-PT network model at depths ranging from 5 to 9 layers. Table 8 shows the performance of the PT segmentation model across various depths. It appears that the size of the input image has an impact on the PT segmentation model, with performance enhancements occurring at a depth of 224. However, increasing the size to 320 or 512 results in a decrease in the model’s performance as the over-deep network structure leads to feature saturation and overfitting. Experiments indicate that the parallel ViT module of the PT model attains optimal performance at 6 layers. Similarly, Table 9 illustrates how depth impacts the performance of the C-PT model. The model achieves better segmentation performance with 7 layers of depth at different scales. However, when the image size is 320, the C-PT model reaches its highest performance at 8 layers of depth. In summary, the model’s modularity improves while the segmentation performance decreases; thus, the PT model employs 6 layers of depth, and the C-PT model uses 7 or 8 layers of depth to acquire the features, which may relieve the model’s optimization issue to a certain degree.

### 4.6. Ablation Experiment

In order to validate the efficiency of the C-PTransformer module, this paper has conducted combined ablation studies in various settings, such as DD and MLP layers.

**Figure 5:**
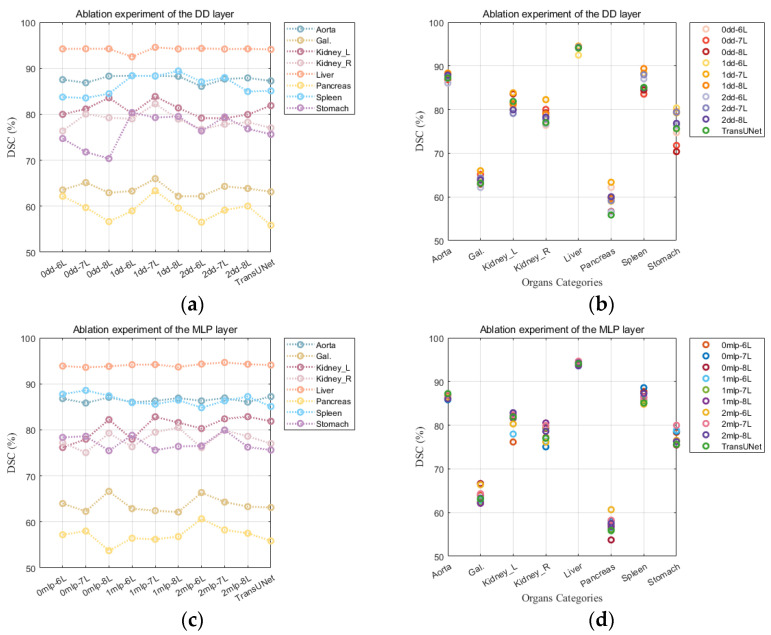
Experimental diagram of ablation of DD and MLP layers. (**a**) DSC performance impact of different DD layers; (**b**) DSC distribution of abdominal 8 organs under different DD layers; (**c**) DSC performance impact of different MLP layers; (**d**) DSC distribution of abdominal 8 organs under different MLP layers.

We examined the impact of utilizing varying quantities of DD layers on the performance of the C-PT model. As shown in Figure 5a, it is evident that C-PT/1dd-7L can achieve a DSC advantage on the Liver, Left Kidney, Right Kidney, Gallbladder, and Pancreas organs. However, altering the number of DD layers to 2 or reducing it to 0 did not improve the DSC performance. In Figure 5b, it is observed that the DD layer does not significantly enhance Liver segmentation, but it does improve Pancreas segmentation significantly. This indicates that the DD layer has the potential to correctly segment challenging targets. Table 10 illustrates that the introduction of the DD layer has resulted in improved average DSC and HD scores as compared with the baseline model. This outcome highlights the efficacy of incorporating the “DD layer and activation function”. In terms of average DSC and HD indices, the C-PT/1DD model outperforms the C-PT/0DD and C-PT/2DD models. The incorporation of the DD layer significantly enhances the feature extraction of the segmentation model. The quantity of parameters and FLOPs are significantly impacted by the DD layer. The 7-layer C-PT/2DD model, when compared with the baseline model, still maintains a competitive advantage concerning the number of parameters and FLOPs. However, an excess of DD layers results in feature saturation within the module, which hinders the model’s convergence and negatively impacts segmentation performance. Therefore, we introduced a 1-layer DD module with a combination of activation functions, resulting in lower computational overhead and improved segmentation performance compared to the serial ViT baseline model.

We investigated the impact of varying numbers of MLP layers on the C-PT model following the addition of an equal number of DD layers. Figure 5c displays that C-PT/0MLP (with only the C-PMHSA component retained) demonstrated excellent DSC performance for Gallbladder and Spleen organ segmentation, while C-PT/2MLP showed superior results for Pancreas and Gallbladder segmentation. In Figure 5d, it is evident that the quantity of MLP layers has a minimal effect on DSC performance improvement for Liver and Aorta segmentation. However, it has a significant influence on segmenting Left Kidney, Right Kidney, and Pancreas organs. Table 11 indicates that C-PT/0MLP and C-PT/1MLP exhibit similar performance in segmentation metrics. The addition of two MLP layers in parallel leads to improved performance, but an excessive number of MLP layers increases the number of parameters and FLOPs, imposing significant computational overhead. Notably, C-PT/1DD introduces only the activation function block, which has a similar computational cost as C-PT/0MLP, yet achieves better segmentation performance than both C-PT/1MLP and C-PT/2MLP. This validates the possibility of improving the feature extraction capacity of the MHSA component, thereby reducing the computational burden of the MLP.

### 4.7. Visualising Results on Synapse Dataset

**Figure 6:**
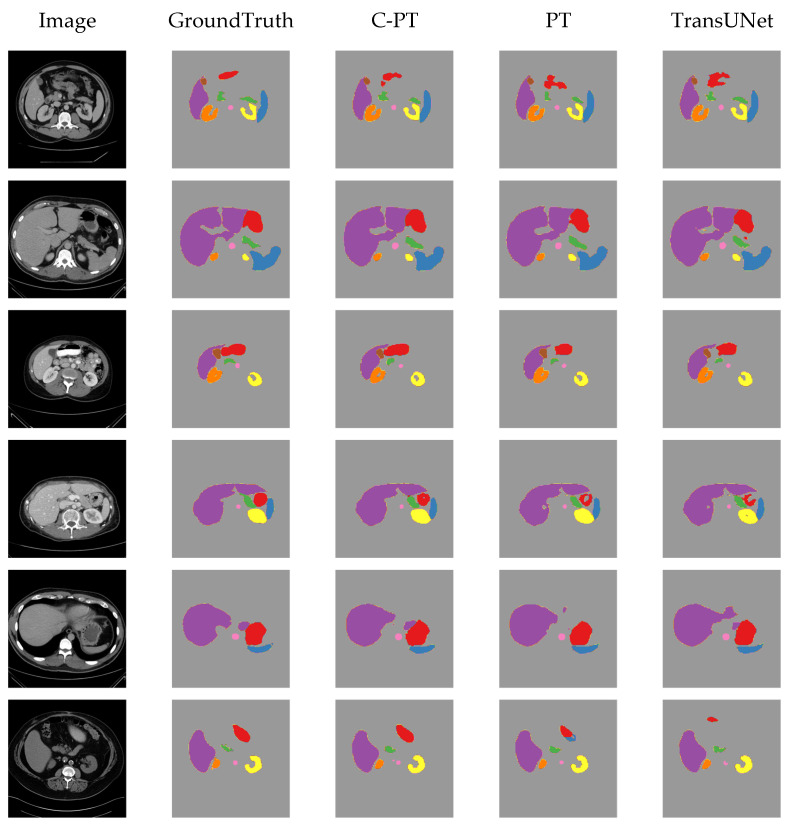
The visualization of model segmentation results on the synapse dataset. Aorta, Gallbladder, Left Kidney, Right Kidney, Liver, Pancreas, Spleen, and Stomach are represented by pink, brown, orange, yellow, purple, green, blue, and red colors, respectively.

Figure 6 displays the visualization outcomes from various methods on the Synapse dataset. The visualization results reveal that our C-PT model outperforms the baseline model in segmenting the Stomach and Liver. Additionally, there is a low probability of actual background regions being predicted as organ areas. This advantage stems from our designed C-PMHSA module, where the output from the left layer guides the right layer to focus on regions containing organs rather than freely learning features. This study indicates that by enhancing the MHSA feature extraction capability and reducing the FFN computational overhead, the method can be modelled more effectively over long distances, leading to improved segmentation results.

## 5. Conclusions

This paper examines the effect of incorporating parallel ViT and C-PT modules to enhance the medical segmentation baseline model, TransUNet, in terms of segmentation efficiency and performance from the perspective of improving ViT. This study proposes the PTransUNet (PT) model and C-PTransUNet (C-PT) model for medical image segmentation. On the basis of the research findings, it can be concluded that:

(1) The PT model demonstrates superior DSC performance compared with the TransUNet baseline model while maintaining the same number of parameters and FLOPs. Additionally, the parallel ViT proves more appropriate for the baseline model than the serial ViT for feature learning at a deeper level.
(2) At an input size of 224, the C-PT model decreases parameter count by 29% and FLOPs by 21.4% as compared with the baseline model, while also improving DSC accuracy by 3.25% and shortening HD edge gap by 10.54 mm compared to the baseline. The C-PT model exhibits superior segmentation performance and higher efficiency than the baseline model employed.
(3) The C-PT module demonstrates improved performance and efficiency when compared to the parallel ViT module within the baseline model. This is attributed to the design of the C-PMHSA and the streamlined MLP. The MHSA block’s feature extraction capability is enhanced to ensure the overall performance of the C-PT module, while the FFN block is replaced with an activation function to reduce the number of parameters and FLOPs of the C-PT module.

This study confirms that the parallel ViT and the proposed C-PT module achieve superior segmentation performance compared to the serial ViT under the baseline model. A future study will examine how the C-PT module can be implemented in 3D medical image segmentation. Moreover, the study will explore the performance benefits of the module by applying it to other network models.

## Figures and Tables

**Table 1:** Network model of the Transformer’s connection method.

Model	Sequential Transformer	Parallel Transformer
TransUNet	√	
Swin-Unet	√	
UNETR	√	
PTransUNet		√
C-PTransUNet		√

**Table 4:** Comparative results of mainstream 2D models on the ACDC dataset. The best results are shown in bold. DSC stands for the higher the better and HD95 for the lower the better. The input image size is uniformly 224.

Methods	Average		RV		Myo		LV	
	DSC↑	HD95↓	DSC↑	HD95↓	DSC↑	HD95↓	DSC↑	HD95↓
R50 U-Net [3]	87.55	-	87.1	-	80.63	-	94.92	-
R50 Att-Unet [48]	86.75	-	87.58	-	79.2	-	93.47	-
VIT CUP [13]	81.45	-	81.46	-	70.71	-	92.18	-
R50 VIT CUP [13]	87.57	-	86.07	-	81.88	-	94.75	-
SwinUNet [6]	90.00	-	88.55	-	85.62	-	95.83	-
MISSFormer [49]	87.90	-	86.36	-	85.75	-	91.59	-
UNETR [7]	87.38	-	88.49	-	82.04	-	91.62	-
CTC-Net [41]	**90.77**	-	**90.09**	-	85.52	-	**96.72**	-
TransUNet	89.59	6.91	88.54	18.11	86.13	1.36	94.12	1.28
Ours ^1^	89.49	6.50	88.16	16.91	86.29	1.32	94.02	1.26
Ours ^2^	90.44	**5.30**	90.02	**13.34**	**86.51**	**1.34**	94.80	**1.22**

^1^ represents the PTransUNet model. ^2^ represents the C-PTransUNet model.— represents that no indicator data were given in the original paper.

**Table 5:** Comparative results of baseline and enhanced models on the ACDC dataset. The best results are shown in bold. Better performance, measured in percentage, follows an increase in values for Accuracy, F1 Score, Sensitivity, and Precision. The input image size is uniformly 224.

**Methods**	**Average**		**RV**		**Myo**		**LV**	
	**Sens↑**	**Prec↑**	**Sens↑**	**Prec↑**	**Sens↑**	**Prec↑**	**Sens↑**	**Prec↑**
TransUNet	87.01	87.16	82.52	**79.58**	85.23	86.48	93.29	95.43
Ours ^1^	86.64	**87.19**	81.84	79.21	85.29	**86.72**	92.79	**95.66**
Ours ^2^	**87.90**	86.84	**83.76**	79.05	**85.89**	86.64	**94.08**	94.82
**Methods**	**Average**		**RV**		**Myo**		**LV**	
	**Acc↑**	**F1-S↑**	**Acc↑**	**F1-S↑**	**Acc↑**	**F1-S↑**	**Acc↑**	**F1-S↑**
TransUNet	99.80	86.71	99.79	80.37	99.73	85.65	99.89	94.13
Ours ^1^	99.80	86.61	99.79	79.99	99.73	85.82	99.89	94.02
Ours ^2^	99.80	**87.08**	99.79	**80.88**	99.73	**86.04**	99.89	**94.32**

^1^ represents the PTransUNet model. ^2^ represents the C-PTransUNet model. Acc, F1-S, Sens, and Prec respectively represent Accuracy, F1 Score, Sensitivity, and Precision.

**Table 6:** Impact of parallelism on the performance of PTransUNet segmentation models.

Models	Size	Parallelism		Average		Params (M)	FLOPs (G)	Train Time
		Branches	Layer	DSC↑	HD↓			
PT/6 × 2	224	2	6	78.35	26.38	88.91	24.73	1:43:03
	320	2	6	81.88	30.17	88.91	50.5	3:33:34
	512	2	6	82.85	28.12	88.91	129.56	10:23:42
PT/4 × 3	224	3	4	78.9	26.53	88.91	24.73	1:42:33
	320	3	4	81.91	25.33	88.91	50.5	3:30:20
	512	3	4	81.84	33.25	88.91	129.56	10:23:45
PT/3 × 4	224	4	3	78.49	25.28	88.91	24.73	1:42:39
	320	4	3	80.96	29.87	88.91	50.5	3:28:56
	512	4	3	74.39	27.48	88.91	129.56	10:28:56

**Table 7:** Impact of parallelism on the performance of C-PTransUNet segmentation models.

Models	Size	Parallelism		Average		Params (M)	FLOPs (G)	Train time
		Branches	Layer	DSC↑	HD↓			
C-PT/ 6 × 2	224	2	6	78.87	24.51	55.17	17.8	1:26:59
	320	2	6	81.2	28.86	55.17	36.37	2:58:21
	512	2	6	80.24	26.07	55.17	93.37	8:58:56
C-PT/ 4 × 3	224	3	4	79.55	26.97	55.16	17.8	1:25:38
	320	3	4	79.03	38.38	55.16	36.35	2:57:19
	512	3	4	75.12	18.17	55.16	93.34	8:58:51
C-PT/ 3 × 4	224	4	3	79.67	25.02	31.48	17.8	1:26:02
	320	4	3	80.58	33.51	31.48	36.36	2:57:53
	512	4	3	82.63	26.11	31.48	93.37	8:58:54

**Table 8:** The impact of depth on the performance of PTransUNet segmentation models.

Model	Size	Depth		Average		Params (M)	FLOPs (G)	Train Time
		Branches	Layer	DSC↑	HD↓			
PT	224	2	5	77.31	31.52	75.39	21.95	1:28:56
	224	2	6	78.35	26.38	88.91	24.73	1:43:03
	224	2	7	79.18	27.82	102.43	27.51	1:51:51
PT	320	2	5	81.11	27.63	75.39	44.82	3:07:48
	320	2	6	81.88	30.17	88.91	50.5	3:33:34
	320	2	7	81.37	23.58	102.43	56.18	3:51:43
PT	512	2	5	80.82	37.47	75.39	114.97	9:09:44
	512	2	6	82.85	28.12	88.91	129.56	10:23:42
	512	2	7	73.66	27.44	102.43	144.14	11:37:46

**Table 9:** The impact of depth on the performance of C-PTransUNet segmentation models.

Model	Size	Depth		Average		Params (M)	FLOPs (G)	Train Time
		Branches	Layer	DSC↑	HD↓			
C-PT/ 6 × 2	224	2	5	78.78	30.36	47.28	16.18	1:19:27
	224	2	6	78.87	24.51	55.17	17.8	1:26:59
	224	2	7	80.73	21.15	63.07	19.43	1:34:25
	224	2	8	79.17	27.52	70.96	21.05	1:41:10
	224	2	9	78.59	28.78	78.86	22.68	1:48:22
C-PT/ 4 × 3	320	2	5	80.47	31.62	47.28	33.04	2:41:18
	320	2	6	81.2	28.86	55.17	36.37	2:58:21
	320	2	7	81.26	29.43	63.07	39.69	3:14:48
	320	2	8	82.66	21.11	70.96	43.01	3:36:37
	320	2	9	80.51	25.91	78.86	46.34	3:52:46
C-PT/ 3 × 4	512	2	5	80.28	28.27	47.28	84.82	8:05:24
	512	2	6	80.24	26.07	55.17	93.37	8:58:56
	512	2	7	81.85	22.32	63.07	101.93	9:52:13
	512	2	8	81.52	23.21	70.96	110.48	10:47:44
	512	2	9	82.1	24.89	78.86	119.03	11:44:54

**Table 10:** The impact of DD layer on the performance of C-PTransUNet segmentation models.

Model	Average		CP-T Block		Block Layer	Params (M)	FLOPs G)
	DSC↑	HD↓	DD Layer	MLP			
C-PT/ 0DD	77.77	29.93	0	f	6	34.89	13.64
	77.8	29.03	0	f	7	39.41	14.57
	77.47	33.91	0	f	8	43.93	15.51
C-PT/ 1DD	78.87	24.51	+1	f	6	55.17	17.8
	80.73	21.15	+1	f	7	63.07	19.43
	79.17	27.52	+1	f	8	70.96	21.05
C-PT/ 2DD	77.29	28.64	+2	f	6	75.45	21.96
	78.69	28.57	+2	f	7	86.72	24.28
	78.24	30.76	+2	f	8	97.99	26.61

Note: 0 represents that no DD layer was used, +1 represents that one DD layer was used, +2 represents that two DD layers were used, and f represents that only the activation function was utilized.

**Table 11:** The impact of MLP layer on the performance of C-PTransUNet segmentation models.

Model	Average		CP-T Block		Block Layer	Params (M)	FLOPs (G)
	DSC↑	HD↓	DD Layer	MLP			
C-PT/ 0MLP	77.66	28.23	+1	0	6	55.16	17.8
	77.48	35.41	+1	0	7	63.06	19.42
	78.2	27.68	+1	0	8	70.95	21.05
C-PT/ 1MLP	77.31	28.88	+1	+1	6	82.19	23.35
	77.82	27.72	+1	+1	7	94.59	25.9
	78.05	27.6	+1	+1	8	106.99	24.45
C-PT/ 2MLP	78.17	31.38	+1	+2	6	109.22	28.9
	79.08	28.14	+1	+2	7	126.13	32.38
	78.25	29.54	+1	+2	8	143.03	35.86

Note: One layer was employed for the DD layer, while for the experiments, MLP utilized layers 0, 1, and 2.

## Data Availability

Two publicly available datasets were used in this manuscript: the Multi-Organ CT Image Seg-mentation Dataset for the MICCAI Multi-Atlas Abdominal Labelling Challenge (Synapse) and the MRI Image Segmentation Dataset for Automated Cardiac Diagnostics Challenge (ACDC). These datasets can be found at https://www.synapse.org/#!Synapse:syn3193805/wiki/217789 (accessed on 5 May 2023), and https://www.creatis.insa-lyon.fr/Challenge/acdc/ (accessed on 5 May 2023), respectively.

## Abbreviations

The following abbreviations are used in this manuscript:

PT	PTransUNet network
C-PT	C-PTransUNet network
CNN	Convolutional neural network
ViT	Vision Transformer
MHSA	Multi-Head Self-Attention
FFN	Feed-Forward Network
DSC	Dice similarity coefficient
HD95	the 95% Hausdorff Distance
FLOPs	Floating point operations per second
MLP	Multilayer Perceptron
LN	Layer Normalization
GELU	Gaussian Error Linear Unit
