# Correction: Wang et al. Cross-Parallel Transformer: Parallel ViT for Medical Image Segmentation. *Sensors* 2023, *23*, 9488

**DOI:** 10.3390/s24020586

**Published:** 2024-01-17

**Authors:** Dong Wang, Zixiang Wang, Ling Chen, Hongfeng Xiao, Bo Yang

**Affiliations:** College of Engineering and Design, Hunan Normal University, Changsha 410081, China; 202120183241@hunnu.edu.cn (D.W.); 202270183474@hunnu.edu.cn (Z.W.); lcheno@hunnu.edu.cn (L.C.); 18374972137@163.com (H.X.)

## Text Correction

There was an error in the original publication [1]. Due to hardware limitations, the single NVIDIA A5000 GPU we used only had 24 GB of memory and was unable to run with an input image of 512 and batch size = 24.

A correction has been made to 4. Experiments and Results, 4.2. Implementation Details, Paragraph 1:

Our experiments were conducted using the PyTorch framework with a single NVIDIA A5000 GPU that has 24 GB of memory. To ensure objective comparison with the baseline TransUNet, we applied the same data augmentation technique as used in the TransUNet model to prevent overfitting. We also set the appropriate input resolution (224 × 224, 320 × 320) and patch size P = 16. The same optimizer and parameters [5], comprising a learning rate of 0.01, momentum of 0.9, weight decay of 1 × 10^−4^, etc., were used for training the model. Based on the TransUNet model, we set the batch size to 24 and the number of training iterations to 14 k for the Synapse dataset [5]. Under the stipulation of maintaining the initial requirements, we preserved the pre-training parameters of ResNet-50 [36] concerning ImageNet [40] in the TransUNet design. For the 12-layer Transformer component’s encoder part, we substituted it with a C-PT block with the most suitable number of layers. We employed some of the ViT pre-training parameters to enhance training effectiveness. Following this, we completed general training to adjust the network weights. We additionally utilized 2D inputs for forecasting, followed by the reconstruction of the model in 3D for assessment of the impact. In particular, all Synapse experiments with 512 input image sizes in this paper were performed with a batch size of 6 and a learning rate of 0.0025, which is different from the TransUNet conditions.

The authors state that the scientific conclusions are unaffected. This correction was approved by the Academic Editor. The original publication has also been updated.
